# Knowledge translation of the HELPinKIDS clinical practice guideline for managing childhood vaccination pain: usability and knowledge uptake of educational materials directed to new parents

**DOI:** 10.1186/1471-2431-13-23

**Published:** 2013-02-08

**Authors:** Anna Taddio, Vibhuti Shah, Eman Leung, Jane Wang, Chaitya Parikh, Sarah Smart, Ross Hetherington, Moshe Ipp, Rebecca Pillai Riddell, Michael Sgro, Aleksandra Jovicic, Linda Franck

**Affiliations:** 1 Clinical Social and Administrative Pharmacy, Leslie Dan Faculty of Pharmacy, University of Toronto, 144 College Street, Toronto, Ontario, M5S 3M2, Canada; 2 Child Health Evaluative Sciences, The Hospital for Sick Children, 555 University Avenue, Toronto, Ontario, M5G 1X8, Canada; 3 Department of Paediatrics, Mount Sinai Hospital, 600 University Avenue, Toronto, Ontario, M5G 1X5, Canada; 4 Knowledge Translation Program, Li Ka Shing Knowledge Institute, St. Michael’s Hospital, 60 Bond Street, Toronto, Ontario, M5B 1W8, Canada; 5 Undergraduate pharmacy division, Leslie Dan Faculty of Pharmacy, University of Toronto, 144 College Street, Toronto, Ontario, M5S 3M2, Canada; 6 Graduate Department of Pharmaceutical Sciences, Leslie Dan Faculty of Pharmacy, University of Toronto, 144, College Street, Toronto, Ontario, Canada, M5S 3M2; 7 Graduate Department of Pharmaceutical Sciences, Leslie Dan Faculty of Pharmacy, University of Toronto, 144, College Street, Toronto, Ontario, M5S 3M2, Canada; 8 AboutKidsHealth, The Hospital for Sick Children, 555 University Avenue, Toronto, Ontario, M5G 1X8, Canada; 9 Department of Paediatrics, The Hospital for Sick Children, 555 University Avenue, Toronto, Ontario, M5G 1X8, Canada; 10 Department of Psychology, Faculty of Health, York University, 4700 Keele Street, Toronto, Ontario, M3J 1P3, Canada; 11 Department of Paediatrics, Keenan Research Centre, Li Ka Shing Knowledge Institute, St. Michael’s Hospital, 60 Bond Street, Toronto, Ontario, Canada, M5B 1W8; 12 Knowledge Translation Program, Li Ka Shing Knowledge Institute, St. Michael’s Hospital 60 Bond Street, Toronto, Ontario, M5B 1W8, Canada; 13 Department of Family Health Care Nursing, University of California, San Francisco, 2 Koret Way, N411F, Box 0606, San Francisco, CA, 94143, USA

**Keywords:** Vaccination, Pain management, Infant/child, Health information, Knowledge translation, Implementation, Parent education

## Abstract

**Background:**

Although numerous evidence-based and feasible interventions are available to treat pain from childhood vaccine injections, evidence indicates that children are not benefitting from this knowledge. Unrelieved vaccination pain puts children at risk for significant long-term harms including the development of needle fears and subsequent health care avoidance behaviours. Parents report that while they want to mitigate vaccination pain in their children, they lack knowledge about how to do so. An evidence-based clinical practice guideline for managing vaccination pain was recently developed in order to address this knowledge-to-care gap. Educational tools (pamphlet and video) for parents were included to facilitate knowledge transfer at the point of care. The objectives of this study were to evaluate usability and effectiveness in terms of knowledge acquisition from the pamphlet and video in parents of newly born infants.

**Methods:**

Mixed methods design. Following heuristic usability evaluation of the pamphlet and video, parents of newborn infants reviewed revised versions of both tools and participated in individual and group interviews and individual knowledge testing. The knowledge test comprised of 10 true/false questions about the effectiveness of various pain management interventions, and was administered at three time points: at baseline, after review of the pamphlet, and after review of the video.

**Results:**

Three overarching themes were identified from the interviews regarding usability of these educational tools: receptivity to learning, accessibility to information, and validity of information. Parents’ performance on the knowledge test improved (p≤0.001) from the baseline phase to after review of the pamphlet, and again from the pamphlet review phase to after review of the video.

**Conclusions:**

Using a robust testing process, we demonstrated usability and conceptual knowledge acquisition from a parent-directed educational pamphlet and video about management of vaccination pain. Future studies are planned to determine the impact of these educational tools when introduced in clinical settings on parent behaviors during infant vaccinations.

## Background

Routine childhood immunization is a proven tool for eradicating and controlling infectious diseases. Despite its key role in maintaining global public health, numerous individuals either refuse or delay immunization [1,2]. One of the well-documented barriers to immunization is pain from the requisite needle puncture or ‘shot.’[3] There are several ways in which injection pain leads to non-compliance. First, either parents or health care providers withhold vaccination in children to avert child suffering [3,4]. Second, children refuse vaccinations themselves. Third, negative experiences with vaccination pain in childhood lead to development of life-long needle fears and associated health care avoidance behaviours in adulthood, including immunization non-compliance [3].

It is estimated that one-quarter of adults are afraid of needles [5]; vaccine non-compliance occurs in upwards of two-thirds of them [5]. Outbreaks of vaccine-preventable diseases have been documented to begin in individuals that refused immunization [1] or, due to reduced herd immunity, among infants too young to be immunized [2]. Thus, the success of immunization programs is compromised, in part, because pain induces avoidance of immunization and sub-optimal coverage rates [6].

Numerous evidence-based and feasible interventions are available to mitigate childhood vaccination pain [7-9]; however, there is low uptake of these interventions in clinical practice, subjecting children to unnecessary pain and suffering and the risk of long-term harms, including immunization non-compliance [10]. Thus, a knowledge-to-care gap exists between what is known about vaccination pain management and what is being done to manage pain during childhood vaccine injections in clinical practice.

According to the Knowledge-to-Action Framework [11], for scientific evidence to be adopted in clinical practice, best-practice guidelines and educational tools are needed. We convened an inter-disciplinary panel from across Canada in 2008 called the Help ELiminate Pain in KIDS (HELPinKIDS) Team in order to address this issue [12]. Using the Knowledge-to-Action Framework [11], the HELPinKIDS Team developed a clinical practice guideline (CPG). Published scientific literature, key informant interviews and discussions with panel members and stakeholder partners, including parents, were used to develop recommendations for 18 pain-relieving interventions [12].

Educational tools (pamphlet and video) directed to parents were developed in collaboration with http://www.aboutkidshealth.ca [The Hospital for Sick Children (SickKids), Toronto, Canada], the primary health information website dedicated to pediatrics, in order to facilitate the adoption of world evidence-based practices at the point of care [13]. Parents were identified as the primary targets for education because they are responsible for all aspects of their children’s care and must acquire the necessary knowledge and skills to optimally care for their children [14]. In previous studies and in stakeholder meetings convened by the HELPinKIDS Team, parents reported a knowledge gap in their education about effective vaccination pain management and a strong desire to learn about ways to reduce pain during their children’s vaccinations [10]. In addition, they reported that this knowledge gap is the major reason for under-utilization of pain-relieving strategies during their children’s vaccine injections [15]. Previous research also demonstrates that when pain-relief is provided to children undergoing vaccine injections, parents experience higher levels of satisfaction with the medical encounter [16]. Teaching and empowering parents therefore has the potential to lead to significant improvements in current pain management practices, vaccine compliance, and health outcomes for children.

The original educational tools developed by HELPinKIDS included information about evidence-based intervention options for children of all ages. An important step in translating research evidence into practice involves adaptation of the knowledge tools to the context of use through an iterative process of obtaining feedback from the different intended end-users and modifying the tool according to their emerging needs [11]. In this study, we continued the process of customizing the educational tools to better suit the needs of intended users. We targeted parents of newborn infants in a hospital setting shortly after birth and used a robust testing process to evaluate: 1) usability of the tools and 2) changes in parents’ uptake of knowledge about evidence-based pain-relieving interventions.

## Methods

### Study design

We employed a mixed methods design. The qualitative component consisted of a usability testing process that was divided into two steps: 1) heuristic usability evaluation of the HELPinKIDS educational tools by a human factors engineer, and 2) three sets of individual and group interviews with parents to validate and improve the usability of the tools. The quantitative component addressed the effectiveness of the tools, and consisted of an independently completed survey of the quality with which information is provided in the educational tools, and an independently completed test of parents’ knowledge about evidence-based pain-relieving interventions.

### Participants and setting

Mothers and fathers present on the postnatal ward (Mother and Baby Unit, Mount Sinai Hospital, MSH) after the delivery of a newborn infant were eligible for participation. Excluded were parents with infants that were born: (1) < 37 weeks gestation (preterm), (2) with major congenital or chromosomal anomalies, (3) admitted to the neonatal intensive care unit, or (4) scheduled for early discharge (within 6 hours of birth). In addition, we excluded mothers with significant psychiatric conditions and parents that were unable to communicate in English, as identified by the charge nurse. A purposeful sample was taken to ensure a broad representation in terms of age, and prior experience with vaccine injections in children. Ethical approval was obtained from the Mount Sinai Hospital Research Ethics Board and all participants signed a written consent form.

### Development and review of educational tools

The original pamphlet developed with the HELPinKIDS CPG consisted of a 2-sided full page sheet describing pain-relieving interventions for use in children of all ages undergoing vaccine injections [12]. It contained coloured pictures on the front side and coloured pictures and written instructions on the reverse side. The video was a 20-minute documentary film that included an overview of the importance of managing vaccination pain and video vignettes of children of different ages undergoing vaccine injections with and without analgesic interventions.

Before gathering input from potential end-users (i.e., new parents) to improve the usability of the tools, a human factors engineer first conducted a heuristic (usability) evaluation of both materials to determine if design elements followed established principles for user interface design [17]. User requirements were identified and translated into tool design features aimed at ensuring that they met performance and end-user goals/needs.

Feedback from this evaluation was incorporated into the development of the version of the pamphlet and video subsequently provided to parents. For the pamphlet, feedback from the human factors engineer led to the following changes: separating information for infants (< 12 months) and children (> 12 months), ordering interventions according to timing prior to vaccine injection (i.e., providing a timeline for each intervention), and removing and revising some of the images and text. For the video, changes included: reducing the duration to 9 minutes; substituting general information about immunization with specific information about pain management interventions, including visual displays about how to implement individual pain-relieving interventions (e.g., how to apply topical anaesthetics); and restricting the information to young children.

Study participants reviewed information on the pamphlet for infants < 12 months only. The pamphlet was revised after each of the three rounds of interviews in order address comments made by parents. No changes were made to the video throughout the study.

### Study procedures

Three sets of interviews were conducted with parents, involving a total of 8 to 21 parents in each. Parents were recruited from the postnatal ward of the hospital after the birth of an infant (and before any vaccinations had been administered to infants) by a member of the research staff that was not involved in their medical care. Interviews were conducted either individually or in groups, in the ward classroom or in parent private rooms, according to parent preferences. Each interview was divided into three phases: baseline, pamphlet review, and video review. The baseline phase included general questions regarding parents’ knowledge and attitudes about childhood vaccination pain. The pamphlet and video phases included review and feedback of the tools by parents. An experienced moderator (EL) facilitated discussion using a semi-structured interview guide:

What do you know about this topic?

● What do you want to know about this topic?

● What information do health care providers believe parents need to know?

● What should parents need to know and be able to do after REVIEWING this material?

● When should parents get this information?

● Where should parents see this information?

● Ask about specific details regarding layout, understandability, attractiveness, content.

● Additional comments?

and audio-recorded the discourse. When mothers and fathers both participated, they were interviewed together, but were able and encouraged to provide individual comments. Parents completed a demographic form at the end of the interview.

The quantitative component consisted of administration of a validated instrument for assessing the health information (Calgary Health Region Evaluation of Health Information, CHREHI) [18], and a knowledge test specifically developed for this study. The CHREHI instrument includes questions about understandability and adequacy of information and was administered after review of the pamphlet and video. The knowledge test includes 10 true/false questions about the effectiveness of various interventions for reducing vaccine injection pain and was administered at baseline, after presentation of the pamphlet, and after presentation of the video (Table 1). Parents rated their level of confidence in their responses to each question using a 5-point Likert scale (very sure, a little sure, neither sure/nor unsure, a little unsure, very unsure). Finally, parents were asked about their intention to use the techniques included in the pamphlet and video to lessen their baby’s pain from vaccine injections using a 5-point Likert scale (very unlikely, unlikely, neither unlikely nor likely/neutral, likely, very likely).

**Table 1:** Knowledge test for vaccination pain management in infants

	Correct response*
1. Giving sugar water can reduce pain and distress.	True
2. Using medicines like acetaminophen (Tylenol, Tempra), or ibuprofen (Advil, Motrin) can reduce pain and distress.	False
3. Putting ice on the skin can reduce pain and distress.	False
4. Breastfeeding can reduce pain and distress.	True
5. Bottle feeding can reduce pain and distress.	True
6. Holding the baby can reduce pain and distress.	True
7. Using numbing (anaesthetic) medicines can reduce pain and distress.	True
8. Distracting the baby can reduce pain and distress.	True
9. Acting calm can reduce pain and distress.	True
10. Rubbing the skin can reduce pain and distress.	False

* Based on HELPinKIDS clinical practice guideline [12].

### Data analysis

Interview recordings were transcribed verbatim and analysed using content analysis [19]. Data collection and analysis occurred simultaneously until saturation of the key emerging themes occurred. The frequency and consistency in which participants indicated categories of responses in the transcripts was used to provide credibility to these categories. Inter-coder reliability between the 2 coders (EL and AJ) was assessed using Kappa statistics (in NVivo 8), and any disagreements (considered as < 90% agreement) were resolved by the 2 coders achieving consensus.

The baseline mean number of correct responses on the knowledge test was compared to post-pamphlet and post-video scores using repeated measures ANOVA. Analyses were repeated including interview group session (1, 2, 3) and parent (mother, father) as covariates in the model. The statistical program SPSS version 20 was used. A p-value < 0.05 was considered significant.

The qualitative component of the study adheres to the RATS guidelines on qualitative research (http://www.biomedcentral.com/ifora/rats).

## Results

The study was conducted between July 7, 2011 and November 2, 2011. Altogether, 33 mothers of newborn infants (some accompanied by their partners) were approached to participate. Seven mothers declined due to being fatigued; however, in 2 instances, the father agreed to participate. In 9 instances, both mothers and fathers participated. Thus, 26 mothers and 11 fathers were included (total sample size = 37). Demographic characteristics of the participants are displayed in Table 2.

**Table 2:** Characteristics of Parents (n=37)

	**Mean (SD) or Frequency (%)**
Age	33 (4)
Mothers	26 (70)
Prior experience with childhood vaccine injections	18 (49)
University education	22 (60)

### Qualitative analysis for usability testing

Overall, three overarching themes related to the tools were identified in the interviews: 1) receptivity to learning, 2) accessibility to information, and 3) validity of information.

#### Receptivity to learning

Parents were overwhelmingly receptive and open to learn about the pain management strategies they could use for their infants during vaccine injections:

“You know, it’s distressing especially before any kind of injection, you know, and yeah, I think anything like this would help. Any kind of information would help.”

“I think everyone should get this and give… it would [be] very useful to me and all the mothers going home.”

#### Accessibility to tools

Parents wanted access to the educational tools. They specified various settings for the information, including hospital, health care provider’s office, and the internet:

“I would have found it useful if I had this before, you know, even in a package that I got from [the hospital].”

“I think that if they have it more in education classes or your new mom’s classes.”

“Definitely at the pediatrician’s office…while you’re there, while you are waiting in the waiting room, you know? You know the secretary can you know definitely hand this out. Have this there. It would be useful because you are waiting anyways.”

“I think it would be handy if it’s on the internet.”

Parents preferred to have the pamphlet and video together rather than one or the other:

“They complement each other, like they go hand in hand. Like you read the pamphlet and then you actually saw it in action in the video.”

“[The video] was very helpful. Especially when they show us how to like apply the anesthetic. That’s something that I learned. And then you actually see what you have written here you actually see it done in practice. So it was easier, it made it more easier for me to understand and appreciate this.”

#### Validity of information

Parents reported that credibility of the information was important to them. Some parents indicated they could trust the information based on the perceived credibility of its source:

“I do trust the information because I mean I see SickKids as an authority on children’s health so when I saw that I feel more comfortable with this.”

Some parents compared the information with prior experience and external information sources (e.g., doctor or nurse):

“Um, most of the suggestions, most of them I’ve heard before so they weren’t a surprise. I wasn’t sure about every answer that I’ve heard that it’s good to give your child…”

“Avoiding the acetaminophen… not been proven to reduce pain at the injection but it could help afterwards to relieve the pain post [injection], cause that’s what my family doctor always tells me to give them [acetaminophen] after they have their injections.”

In addition, some parents reported they would seek confirmation that the information is accurate before implementing it for vaccine injections:

“I would probably ask [my doctor], yeah. I would probably, you know, say, like I don’t think that … the [acetaminophen] before the injection would be useful. Like what do you think? You know, I would probably ask for a second opinion/ask for their opinion.”

#### Changes to organization and content of the pamphlet

**Figure 1:**
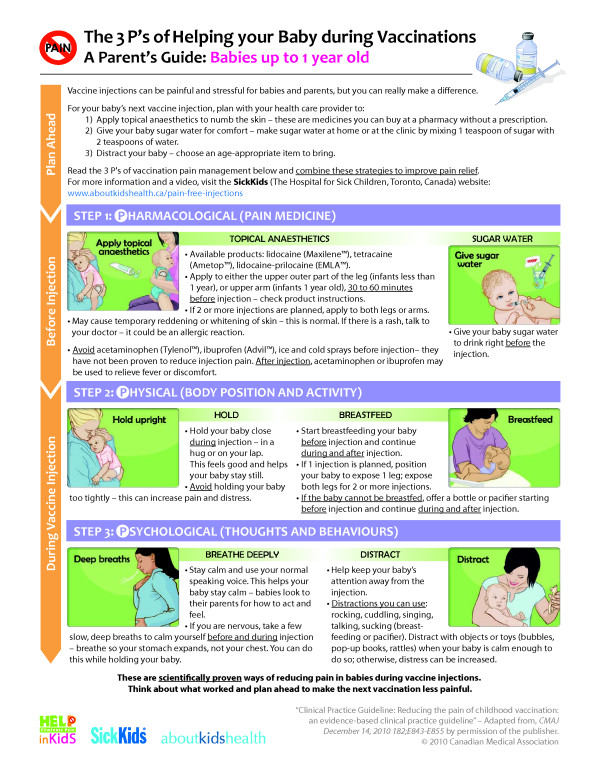
Final version of pamphlet.

Parents reported that they were generally satisfied with the pamphlet; that it was easy to follow, and clear in terms of which methods were effective and ineffective. After each set of interviews, iterative changes were made to improve the visual and textual information, according to the principles of user-centered design. After the first set of interviews, the changes made included: 1) confining information on one side of the page to reduce redundancy and the need to turn the page; 2) re-organizing information according to preparatory activities that are performed ahead of time and pain-relieving activities that are performed on the day of vaccination; and 3) adding the SickKids’ website address for all of the HELPinKIDS resources to improve perceptions of the credibility of the information. In order to accommodate the changes, some textual explanations were shortened or deleted. After the second set of interviews, the following changes were made: 1) the use of colour was reduced to minimize perceptions that the pamphlet was cluttered; 2) some words were underlined to bring attention to them; and 3) the specific names of commercially available topical anesthetic products were included to facilitate acquisition by parents. After the third set of focus interviews, final edits were made to the pamphlet by AboutKidsHealth, and included; improving clarity and quality of images, and harmonizing the punctuation and writing style (Figure 1). The final version of the pamphlet is shown in: http://www.aboutkidshealth.ca/En/HealthAZ/TestsAndTreatments/GivingMedication/Pages/Pain-Free-Injections.aspx.

#### Feedback about the video

Parents reported they were generally satisfied with the content of the video. Specific suggestions for improvement focused mainly on increasing the speed of presentation, reducing repetition of concepts, and harmonizing phraseology and/or re-ordering content, in order to address perceptions that the video was too long and/or repetitive and that word descriptions and/or the order of presentation was inconsistent with the pamphlet. These suggestions were used to revise the video. The video is available on YouTube channel: http://www.youtube.com/watch?v=jxnDc2PxGUc.

### Quantitative analysis – structured feedback and conceptual knowledge

Responses to the CHREHI survey are shown in Table 3. The majority of parents reported they understood all of the information in the pamphlet and video, and that the amount of information was adequate in terms of breadth and depth.

The knowledge test scores for the baseline, post-pamphlet, and post-video phases are shown in Table 4. The mean number of correct responses increased from baseline to post-pamphlet, and from post-pamphlet to post-video; p < 0.001 and p=0.012, respectively. If only answers whereby parents reported both the correct response and complete certainty in their level of confidence regarding their response were included, then the score increased significantly between baseline and post-pamphlet, and between post-pamphlet and post-video; p < 0.001 and p=0.001, respectively. There was no significant effect of either interview group session (1, 2, 3) or parent that participated (mother vs. father); p > 0.05 for both analyses.

**Table 3:** Responses to calgary health region evaluation of health information instrument (n=37)

	**Understood all of the information in pamphlet (%)**	**Adequate information in pamphlet (%)**	**Understood all of the information in video (%)**	**Adequate information in video (%)**
Frequency, (%)	29	29	32	30
	(78)	(78)	(87)	(81)

All parents reported they intended to act on the information included in the pamphlet and the video, with over 80% indicating they were ‘very likely’ to do so.

## Discussion

Over three decades of research has led to the discovery of numerous evidence-based and feasible treatments for the management of pain during childhood vaccine injections [7-9]. Despite this evidence, few children are benefitting from this scientific knowledge [10,15]. The practice of performing vaccine injections in children without analgesia is associated with significant harms, including the development of life-long needle fears and immunization non-compliance [3]. As part of the first step in addressing this important knowledge-to-care gap, we developed a CPG and educational tools for parents to present research knowledge in a clear, concise, and user-friendly format. [12] According to the Knowledge-to-Action Framework [11], the development of high-quality educational tools as an intervention to promote the adoption of the CPG requires the inclusion of research evidence as well as customization of the evidence to the local context through an iterative process of obtaining feedback from potential users and modifying the tools according to the emerging needs of end-users. In this study, we adapted the educational tools to the needs of new parents and evaluated the usability of the tools and the uptake of knowledge as a result of the tools.

The results demonstrated that parents have a keen interest in learning about pain-relieving interventions for infant vaccinations. They identified a variety of methods of dissemination of this education to better reach new parents, including; parent classes, birthing hospitals, doctor’s offices, and the internet. In addition, they expressed a preference to have access to both the pamphlet and video because they viewed them as complimentary. With respect to the content of the tools, parents reported that they understood the information and it met their needs in terms of breadth and depth. Furthermore, parents reported that they intended to use the information in the pamphlet and video for their infant’s vaccine injections.

These findings are consistent with previous studies demonstrating that parents are concerned about a child’s pain during vaccinations [20] and that they have a strong desire to mitigate vaccination pain [10,15]. Therefore, the current practice of under-treating pain during routine childhood vaccinations cannot be attributed to parent apathy about pain. The major barrier to routine pain management, as identified by parents themselves, is a lack of knowledge about evidence-based interventions [10,15].

In addition, significant improvement in parent knowledge about pain management strategies and in confidence level in knowledge occurred after exposure to the educational tools. Parents were largely uncertain about evidence-based pain management options prior to reviewing the educational tools. There was an increase of 250% in the number of responses to the knowledge test that were both correct and whereby parents were completely sure, with significant increases occurring between the baseline and post-pamphlet phases and also between the post-pamphlet and post-video phases. Importantly, evaluation of parent knowledge uptake considered not only correct responses but also level of certainty in responses, as individuals would not be expected to act on their knowledge unless they are confident in it.

We directed the educational tools to new parents because they are the primary stakeholders involved in childhood vaccination and because education is routinely provided to parents around the time of delivery of an infant, thus opportunity exists to incorporate education about vaccine injection pain management within current hospital education programs provided to families of newborn infants. In addition, teaching new parents about pain is the most efficient method of ensuring that children will receive consistent pain management over time and across all medical settings and for all medical procedures encountered, which is key to creating an environment that promotes healthy child development (i.e., is free from harm). A growing body of literature demonstrates the effectiveness of parent training interventions for developing skills necessary to promote optimal infant and child development, including emotional, social and cognitive development [21,22].

**Table 4:** Parent knowledge test scores (n=37)

	**Baseline**	**After pamphlet**	**After video**	P-value (baseline to pamphlet)*	P-value (pamphlet to video)*
Correct	5.4 (1.7)	8.1 (1.2)	8.5 (0.8)	< 0.001	0.012
Correct & Sure	2.0 (1.9)	6.1 (2.1)	7.2 (1.6)	< 0.001	0.001

Values are mean number of correct responses out of 10 (standard deviation, SD).

* RM ANOVA.

The feedback from stakeholders at our HELPinKIDS’ meetings, including parents, clinicians, and policy makers, suggested: 1) a multi-modal approach for parent education, and 2) the use of flexible and portable education formats. Specifically, written information supplemented with pictures and a video with vignettes of children undergoing vaccination with the pain-relieving interventions promoted by the CPG was deemed as vital components of an effective teaching intervention. To this end, a pamphlet and video were developed to promote the adoption of the CPG. These tools allow for a multitude of education formats, including; self-administered, group-based or individually-administered [23]. The results from our previous qualitative and quantitative studies conducted in the same setting and population informed decisions regarding the content and presentation of the educational tools [10,15], that is, they were derived from the learning needs identified by new parents in this setting. The findings of the present study verify that both tools together are optimal to parent training because: 1) parents expressed a preference to have access to both tools, and 2) uptake of knowledge was greater when both tools were used together. It is important to note that both the pamphlet and video focussed on actions parents can undertake to reduce pain during their infants’ vaccine injections and that parents reported that they intended to implement the knowledge at future vaccine injections.

We observed that some knowledge test questions were frequently answered incorrectly by parents, even after review of the pamphlet and video. These questions related to the effectiveness of oral analgesics, rubbing the skin (i.e., tactile stimulation), applying ice, and bottle-feeding. Since information about these interventions was not prominent and in some cases was not even included in either the pamphlet or video, there was no expectation that parents would score perfectly. A priori, a decision was made to emphasize effective rather than ineffective or unproven pain management strategies and to include questions about ineffective strategies to reduce responder bias.

Although we targeted parents, clinicians constitute important knowledge transfer targets for vaccine injection pain management. Prior research by our group demonstrates that parents seek endorsement by health care providers for pain management strategies [10,15]. This notion was confirmed in two ways in the present study. Firstly, parents reported they planned to consult their doctor about the information before using it. Secondly, parents indicated that the pamphlet and video should be available in their doctor’s office. Efforts to improve current pain management practices must therefore also include clinicians to support and facilitate parent efforts to mitigate their child(ren)’s pain. We are currently conducting parallel studies of clinician-directed educational tools in different practice settings in order to ensure that their needs are being met.

Although we demonstrated interest and knowledge acquisition from our educational materials in our study sample, the immediate post-partum period may not be the optimal time for learning about this information because it will not be acted upon by parents until two months later, the age of initiation of an infant’s primary immunization series in many geographical regions. There is also the possibility that some parents may feel fatigued or overwhelmed by the birth of their infant and added responsibilities, and therefore not receptive to learning about this information. In both cases, however, having the information or knowing about how/where to access the information in the future (e.g., internet) may be sufficient as parents can self-administer the education when they are ready to learn.

Strengths of the study include the setting and design. First, the study setting, MSH, afforded us with the opportunity to recruit parents from a large catchment area as it draws patients from the most populated metropolitan area in Canada (over 5.7 million inhabitants). This facilitated inclusion of parents with diverse cultural and ethnic backgrounds, perspectives and practices. In addition, there was ample time for parents to participate in the study since they typically stay in hospital for over 24 hours after the delivery of an infant. The timing of information was synchronized with other educational programs offered to new parents, and takes advantage of their information-seeking needs and motivation for learning. Finally, both parents were included, rather than mothers alone.

Second, the study design included an in-depth exploration of the usability of the educational tools and objective measures of knowledge uptake derived from them. The qualitative component included robust usability testing process, which consisted of a heuristic evaluation and interviews with end-users (i.e., parents) to validate and improve the material based on usability principles. The involvement of the end-users (i.e., parents) in this process improves the probability that it will lead to subsequent changes in behavior [11]. The quantitative component included evaluation of parent knowledge about effective pain-relieving interventions. Demonstrating knowledge acquisition ensures that the educational tools are sufficiently effective for diffusion of knowledge in the relevant user group [11].

## Conclusions

We found that an educational pamphlet and video about infant vaccine injection pain management were welcomed by new parents and improved knowledge about evidence-based pain-relieving interventions. We are now planning studies to evaluate knowledge use from the educational tools, including: utilization of pain-relieving interventions during infant vaccination and whether this results in reduced infant pain experience; and parent and health care provider satisfaction with the immunization experience.

## Competing interests

Dr. Taddio has received research funding from Pfizer and study supplies from Natus and Ferndale, and has consulted for Archimedes.

## Authors’ contributions

AT was involved in conception and design, acquisition of data, analysis and interpretation of data, drafting of manuscript, critical revision, funding, administrative, technical and material support and supervision. AT had full access to all the data in the study and takes responsibility for the integrity of the data and the accuracy of the data analysis. VS was involved in conception and design, acquisition of data, analysis and interpretation of data, critical revision of the manuscript, and administrative support. EL was involved in conception and design, acquisition of data, analysis and interpretation of data, and drafting of manuscript. LF, RH, and MI were involved in conception and design, analysis and interpretation of data, and critical revision of the manuscript. RPR and MS were involved in analysis and interpretation of data, and critical revision of the manuscript. AJ was involved in analysis and interpretation of data, and critical revision of the manuscript. JW, CP, and SS were involved in acquisition of data, analysis and interpretation of data, and critical revision of the manuscript. All authors read and approved the final manuscript.

## Funding

Funding for this study was obtained from a knowledge translation operating grant awarded by the Canadian Institutes of Health Research (CIHR), fund number 111411.

## Pre-publication history

The pre-publication history for this paper can be accessed here:

http://www.biomedcentral.com/1471-2431/13/23/prepub
